# Contribution to Knowledge on Bioapatites: Does Mg Level Reflect the Organic Matter and Water Contents of Enamel?

**DOI:** 10.3390/ijms242115974

**Published:** 2023-11-04

**Authors:** Agnieszka Lasota, Andrzej Kuczumow, Mieczysław Gorzelak, Tomasz Blicharski, Joanna Niezbecka-Zając, Karolina Turżańska, Anna Szabelska, Michał Łobacz, Karolina Wiszumirska, Marek Wieruszewski, Maciej Jarzębski, Mirosław Jabłoński

**Affiliations:** 1 Department of Maxillary Orthopaedics, Medical University of Lublin, Chodźki 6, 20-093 Lublin, Poland; agnieszka.lasota@umlub.pl; 2 Laboratory 196, Radawiec Duży 196, 21-030 Motycz, Poland; andrzej.kuczumow@gmail.com; 3 Clinic of Rehabilitation and Orthopedics, Medical University of Lublin, 20-090 Lublin, Poland; b.leszczynska@umlub.pl (M.G.); tomasz.blicharski@umlub.pl (T.B.); joanna.niezbecka@umlub.pl (J.N.-Z.); karolina.turzanska@umlub.pl (K.T.); miroslaw.jablonski@umlub.pl (M.J.); 4 Department of Dental Techniques with the Lab of Modern Technologies, Medical University of Lublin, Chodźki 6, 20-093 Lublin, Poland; anna.szabelska@umlub.pl; 5 Chair and Department of Oral Surgery, Medical University of Lublin, Chodźki 6, 20-093 Lublin, Poland; michal.lobacz@umlub.pl; 6 Institute of Quality Science, Department of Industrial Products and Packaging Quality, Poznan University of Economics and Business, Al. Niepodległosci 10, 61-875 Poznan, Poland; karolina.wiszumirska@ue.poznan.pl; 7 Department Mechanical Wood Technology, Faculty of Forestry and Wood Technology, Poznan University of Life Sciences, Wojska Polskiego 28, 60-637 Poznan, Poland; marek.wieruszewski@up.poznan.pl; 8 Department of Physics and Biophysics, Faculty of Food Science and Nutrition, Poznań University of Life Sciences, Wojska Polskiego 38/42, 60-637 Poznan, Poland

**Keywords:** bioapatite, animal and human tooth enamel, Mg, water contents, organic matter contents

## Abstract

The matter constituting the enamels of four types of organisms was studied. The variability of the ions was presented in molar units. It was proven that the changes in water contents of the enamel are significantly positively related to changes in Mg; inversely, there is also a strong connection with changes in Ca and P, the main components of bioapatite. The variability in the organic matter has the same strong and positive characteristics and is also coupled with changes in Mg contents. Amelogenins in organic matter, which synthesize enamel rods, likely have a role in adjusting the amount of Mg, thus establishing the amount of organic matter and water in the whole enamel; this adjustment occurs through an unknown mechanism. Ca, P, Mg, and Cl ions, as well as organic matter and water, participate in the main circulation cycle of bioapatites. The selection of variations in the composition of bioapatite occurs only along particular trajectories, where the energy of transformation linearly depends on the following factors: changes in the crystallographic d parameter; the increase in the volume, V, of the crystallographic cell; the momentum transfer, which is indirectly expressed by ΔsinΘ value. To our knowledge, these findings are novel in the literature. The obtained results indicate the different chemical and crystallographic affinities of the enamels of selected animals to the human ones. This is essential when animal bioapatites are transformed into dentistic or medical substitutes for the hard tissues. Moreover, the role of Mg is shown to control the amount of water in the apatite and in detecting organic matter in the enamels.

## 1. Introduction

Hydroxyapatite is a component of numerous biomaterials serving as substitutes/fillings in relation to damaged original tissue [1]. Parts of such hydroxyapatites can be supplied from natural sources, such as the bones of different animals [2,3,4,5]. One approach to developing apatite-based materials is reducing their particle/crystallite size. Reducing this scale in of apatites to nanometer scale provides structures that are similar to natural bone in both scale and chemical composition [6]. Furthermore, nanohydroxyapatite (Nano-HA) has been widely investigated due to other potential biomedical applications, such as in drug carriers, as an inhibitor of the growth of various tumor cells [7], and in surface coating [8]. It should be noted that, nowadays, apatites play an essential role in developing composite materials. Mechanical tests performed by Machalowski et al. [9] showed that the addition of 5% chitin nanofibers into HA increased the composite compression strength by 10 times (compared to pure HA used as a reference sample). Compositions based on an HA with titanium are of high interest for bone regeneration. Results presented by Niespodziana et al. [10] showed that titanium nanocomposite doped with 10 vol% HA was more corrosion-resistant than microcrystalline titanium. Alloys other than titania-based metallic alloys have been studied due to their potential biomedical applications. Kowalski et al. [11] doped HA with silver and added it to a Mg–1Zn–1Mn–0.3Zr alloy to fabricate ultrafine-grained metal matrix composites. Khatkar [12] highlighted the important role of Nano-HA as a potential material for reinforcements in magnesium-based metal matrix composites for biomedical applications, primarily due to the similarities in the density of magnesium and human tissue. A lot of studies have focused on 3D-printable biocompatible materials that can be used for targeted personal implant preparation. Wang et al. [13] reported that Nano-HA inserted into a polylactide (PLA) matrix was successfully printed using a deposition modeling (FDM) technique. The composite showed good biocompatibility and osteogenic induction. One “out of the box” solution for hydroxyapatite-based materials was biomimetic toothpaste, as proposed by Florea et al. [14]. The application of their system might be helpful with treatment of tooth decay through enamel remineralization. 

Carbonated hydroxyapatite [15,16], one of the most important members of the chemical species called apatites [17], is the basic hard material of the tooth enamel. It covers some 96% of the total mass of enamel. Some more or less equal amounts of organic matter and water supplement the composition. Despite apparently scarce contents, both of the above components play essential roles in the enamel, differentiating this material from its mineral counterpart. An organic matrix composed of proteins [18], such as amelogenin [19,20,21], enamelin, ameloblasts, and tuftelin, plays an important function in forming the crystalline part of tooth enamel [22]. Biological hydroxyapatite is not a pure compound, but is saturated with carbonates, sodium, chlorine, magnesium, and, to a minor degree, with fluorine and strontium; in addition, some trace elements (K, Fe, and Zn) are present as well. Their role has been extensively studied for years; however, it has not been fully explained until now [23,24,25]. The spatial distribution of elements inside enamel has been intensively studied, with proven regularity and stoichiometry in the direction: from the dental–enamel junction to the enamel–air boundary [26,27,28]. It became clear that the presence of water, organic matter, and minor elements shaped the specific features of the enamel, differentiating it from pure hydroxyapatite.

This study paid attention to the role of Mg in bioapatites. The involvement of Mg in apatite has been widely studied; together with carbonate ions, it seems to exert greater influence on the host structure and physicochemical features than the relatively scarce chemical contents would indicate. Many authors point out that the preferred position of Mg is the Mg(II) location [29]. Some studies have addressed the growing hydration of the samples joined with the Mg enrichment of apatites [30]. The meaningful crystallographic transformation of apatite into β-tricalcium phosphate (β-TCP) occurs under the influence of small amounts of Mg and elevated temperatures [31,32,33].

Nevertheless, the role of Mg in the formation and behavior of teeth seems to be very obscure and not very strictly quantified. Elucidating more information in this field is evidently more important if we are planning to design materials which can act as a substitute for decaying hard tissue.

Nevertheless, a relatively small number of papers address comparisons between bioapatites derived from different animals to find the one which can be most easily tailored to human needs [34]. Such studies concern one of the most promising fields of production of prosthetic materials through transformation of animal-originated raw materials; of course, all keep in mind the danger of potential contamination, e.g., by prions [35]. 

In parallel, intensive studies have been performed on totally artificial syntheses of bone and teeth substitutes [36].

The main aims of this study can be defined as follows: (1) to review the common features of bioapatites belonging to the tooth enamel of different animals; (2) to show the similarities and differences between them; (3) to search for interrelations between the ions in enamel; (4) to reveal the possible chemical and crystallographic trajectories of mutual transformations of bioapatites; (5) to determine whether the conclusions raised for different animals can be transferred into the human case. In this study, we manage to prove that the substances under scrutiny belong to a single series of isomorphic compounds.

Studies on animal organisms are important; this is because of the potential use of animal-derived materials, and because such provisional experiments cannot be carried out with human subjects. Moreover, such studies are important because we must trace the generalized trends that are present in both human and animal organisms. Our investigation is aligned with the latter option.

## 2. Results

**Figure 1:**
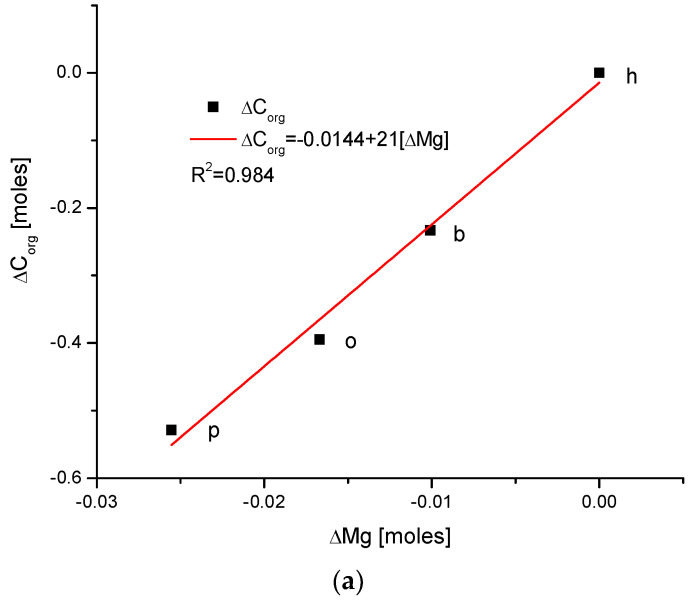

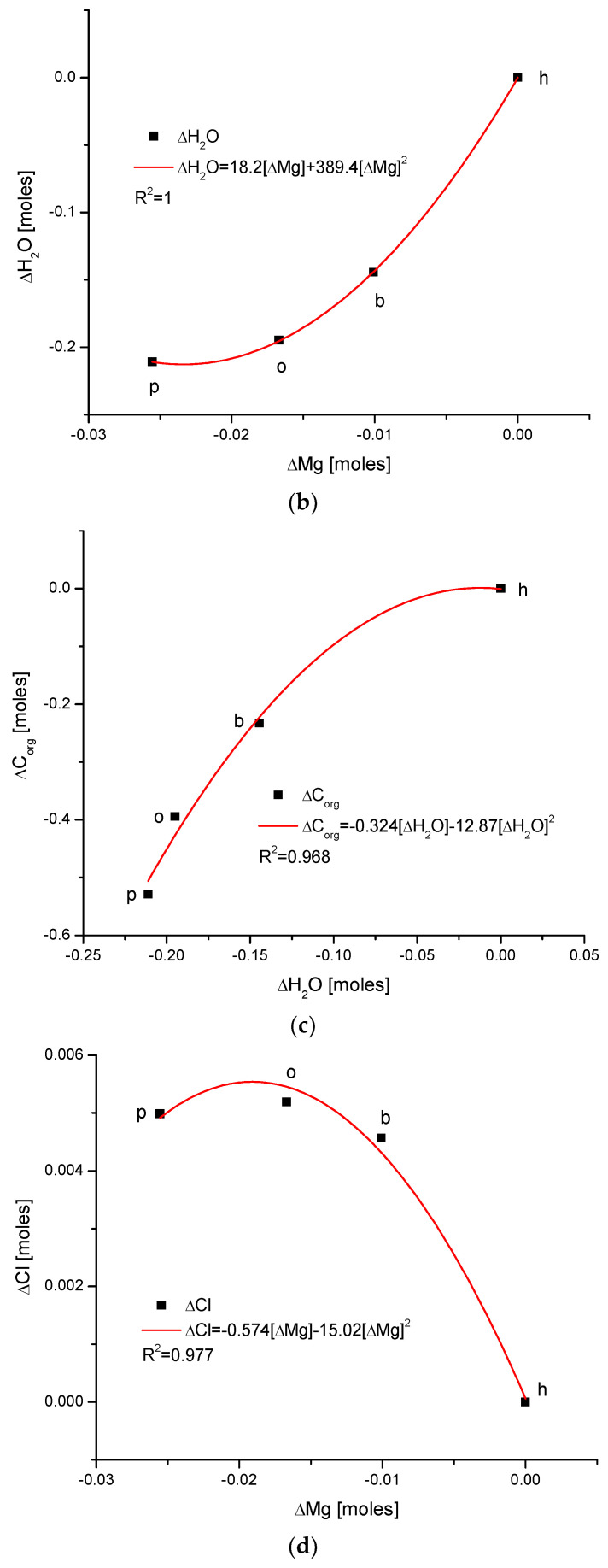
(**a**) Visual representations of the very simple and clear relationships between the change in Mg and the organic matter contents in the enamel of 4 different species; (**b**) strong parabolic relationship between ΔMg and water contents; (**c**) very regular correlation between changes in amounts of water and organic matter; (**d**) somewhat less strong inverted parabolic relationship between changes in ΔCl and ΔMg contents. Note: h—human enamel; b—bovine enamel; o—ovine enamel; p—porcine enamel.

The obtained results will be presented on consecutive plots, to present the data visually. The data concerning different animals are joined in the figures using lines; we note that we are aware that there is sometimes no functional or real dependence between the points. Nevertheless, for the first reason, it is easier to have the lines leading the eyes, and for the second reason, the relationships between the pairs of numbers are significant to note in many cases. The first results concern a comparison with the changes in amount of Mg because we became aware of the meaningful role of this element in the total composition of the tissue [37]; the significance of Mg is not proportional to its relatively small content. We determined the simple correlations between the differences in the contents of different chemical species expressed in moles presented in Figure 1.

**Figure 2:**
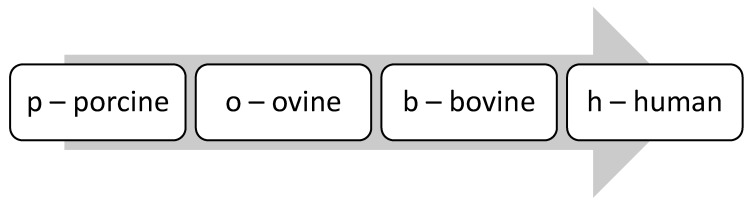
Observe the uniform sequence of the correlations between pairs of chemical elements in the enamels of selected organisms: p–o–b–h.

**Figure 3:**
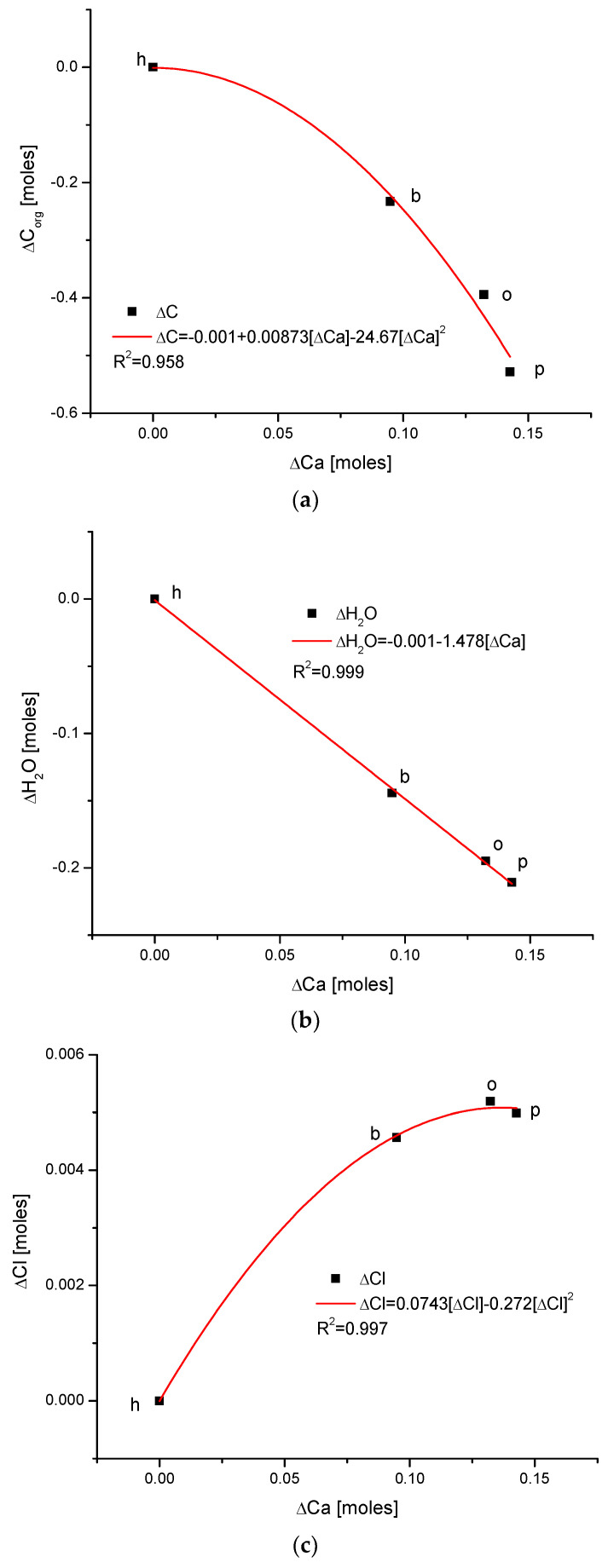
(**a**) The fine inverted correlation between the change in Ca and organic matter contents in the enamel of 4 different species; (**b**) strong straight line inverted relationship between ΔCa and water contents; (**c**) very good parabolic relationship between changes in ΔCl and ΔCa contents.

**Figure 4:**
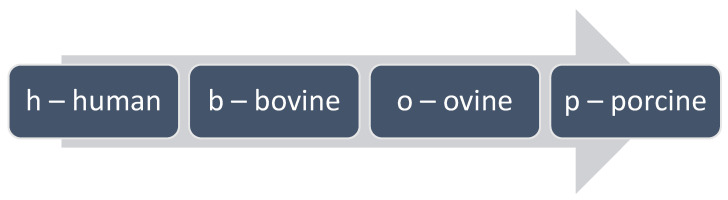
Observe in Figure 3 the uniform sequence between differences of molar contents of different chemical species in different organisms: h–b–o–p.

There exists a strong linear and positive correlation between the growth in contents of organic matter and magnesium (see simple graph in Figure 2 showing the direction of chemical variability). It appears that they are coupled; if the organic matter is present in greater amount, then the bioapatite is more saturated with magnesium. It is important to be aware that the mole value of the organic matter is not reflective of the real value of the moles in the organic substances; this value is reflective of the recalculation of the amounts of the organic carbon atoms for the moles. Similarly, the involvement of water is also linked with the presence of magnesium in the same way, i.e., directly proportional. The relationship is, in this case, more complicated, since it obeys the paraboloidal rule but is still very strong (R^2^ = 1). Next, we can observe a strictly regular, paraboloidal relationship between the water and the organic matter contents—those nonmineral components are mutually dependent in a quantitative manner. All the mentioned relationships have positive correlations, which is very important from the point of view of Mg’s role in the formation of enamel. Moreover, changes in Mg contents correspond to the well-correlated changes in Cl concentrations, which were previously and independently indicated in our earlier papers [26,38] and was found to have important implications for the ordered ion exchanges in enamel (see Figure 3 and Figure 4).

Here, we must provide some comments on the results. They are very meaningful—the strict functional relationships testify that the results concerning four different species are related each other; they obey strong mathematical descriptions and they testify that the investigations published in [25,34] were extremely accurate.

Another series of correlations was found between the changes in calcium contents and the variability of other chemical species. The results are significant, since Ca is one of the two most important elements in apatites.

The relationships that are seen in the changes in Ca contents resemble the information in Figure 1 for changes in Mg. The change in organic carbon contents is clearly paraboloidal, with an impressive coefficient of determination, R^2^, which was close to 1. Even more indicative is the relationship between growth in Ca contents and the decline in water concentration in the bioapatite. This dependence is linear, with an R^2^ near 1. Both dependencies are negatively correlated, contrary to the situation with Mg. The connection between the enrichment in Cl and the growth in Ca contents is very clear and of paraboloidal character; however, the connection if somewhat less strict than in the case of Mg.

**Figure 5:**
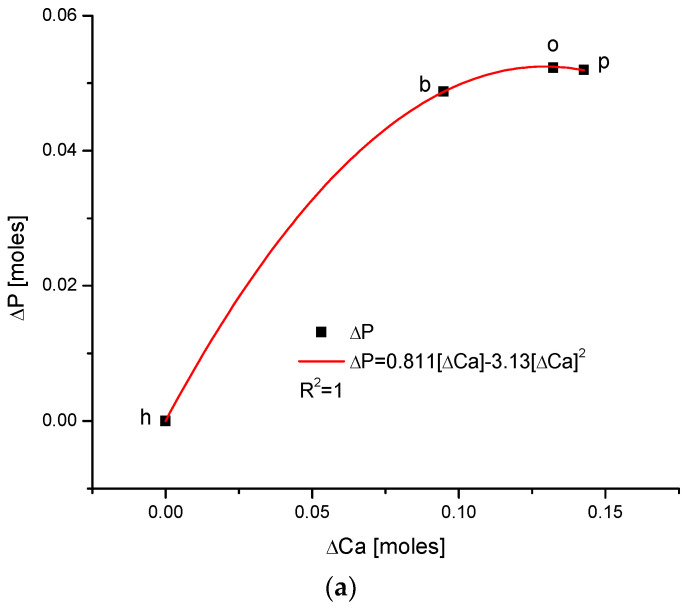

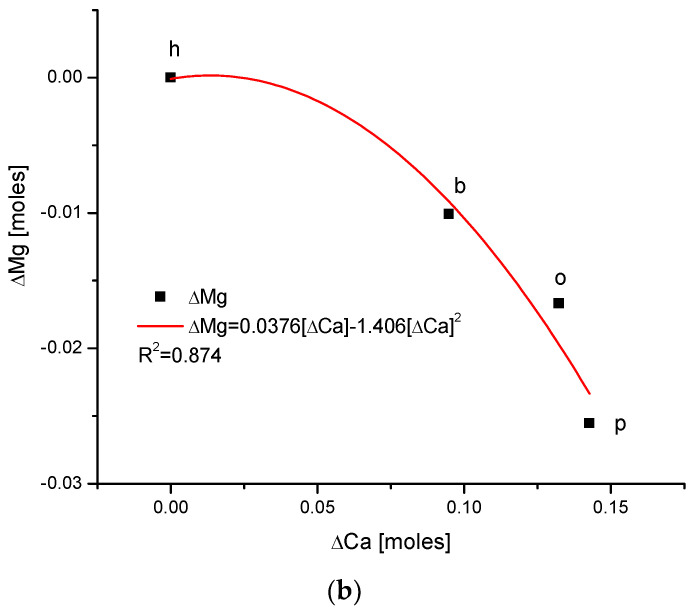
(**a**) Strong parabolic relationship between ΔP and ΔCa; (**b**) the parabolic correlation between changes in ΔMg and ΔCa contents, important in the context of Figure 1 and Figure 3. The sequence is h–b–o–p, as presented in Figure 4.

As one could expect, the relationship between the growth of the two main components of bioapatite is positively correlated in a paraboloidal manner, not a linear one, with the coefficient of determination, R^2^, being equal to 1. The inverted correlation is valid for the ΔMg and ΔCa pair (Figure 5). It supplements the image of all the correlations observed for the enamels of four organisms.

**Figure 6:**
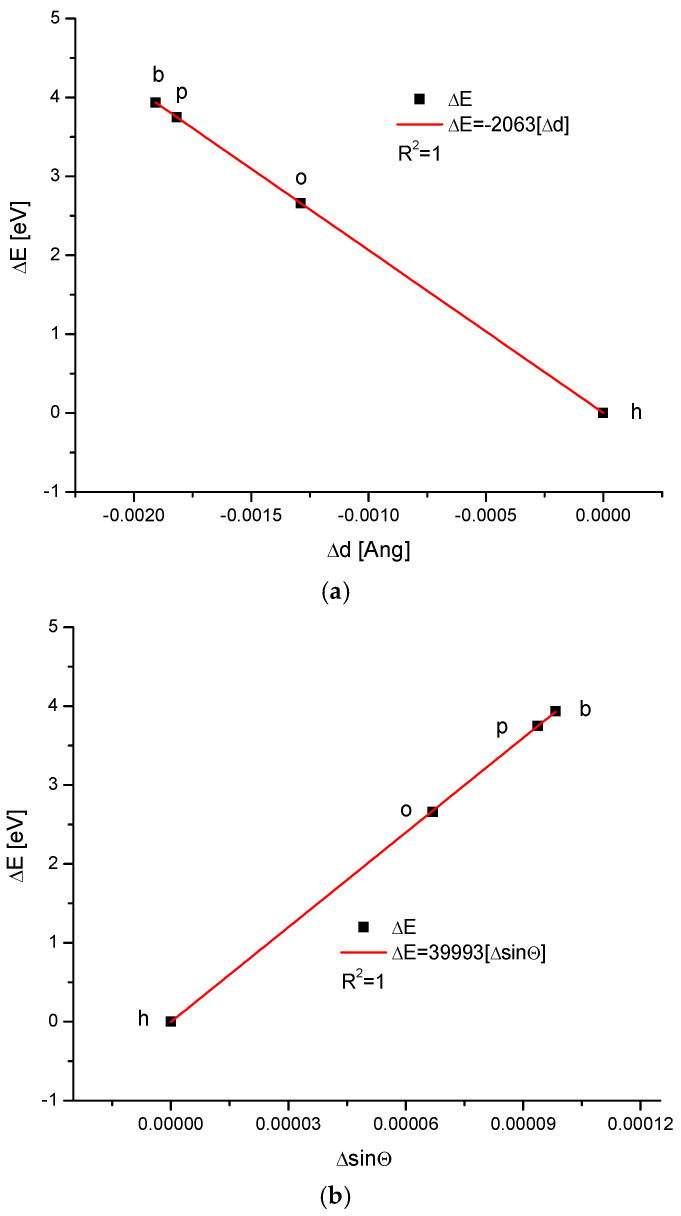
Energy changes connected with (**a**) the change in universal crystallographic d (111) dimension; (**b**) the change in sinΘ value. See the crystallography-based sequence: h–o–p–b. This is slightly different than the one supported by the chemical results.

We applied our methodology to calculate the energy of crystallographic transformations [39] using data from Ortiz-Ruiz et al. [40]. The energy changes resulting from the change in the universal crystallographic dimension d for the configuration (111) or the change in sinΘ are presented in Figure 6. The coupling of the variables is extraordinary each time, with coefficients of determination equal to 1; these findings suggest that the relevant relationships are really functional ones. We must pay attention to the fact that the values of the numerical coefficients in the below equations show that the substances with the same coefficients belong to the same isomorphic series. Equations (1) and (2) show the energetic changes, ΔE:

ΔE = −2063Δd(1)

ΔE = 39,993ΔsinΘ(2)

This be transformed into an instrumental dependence that is valid for X-ray diffraction (XRD):

Δd = −19.3858 ΔsinΘ (3)

## 3. Discussion

Previous studies performed by Khurshid et al. [41] highlighted that hydroxyapatite crystals provide a major contribution to the chemical structure of natural enamel and dentin. Nano-HA composites are classified as emerging materials for dental applications [42]. However, the research is mostly focused on mechanical studies as well as biocompatibility studies without deeper concerns about the elemental composition of apatite. 

Our analysis shows clearly that the presence of Mg is closely coupled with the arrival of water in the apatite of the tooth enamel. The contents of the organic matter are positively correlated with the presence of this element as well. Thus, the quantification of Mg should be equivalent to the quantification of organic matter and water in the enamel. This finding must be taken into account if somebody plans to synthesize artificial bioapatite by introduction of Mg into the apatite matrix, or, alternatively, if they plan to saturate apatites with a proper amount of water. This question was earlier considered in a qualitative study by Rossi et al. [43]. Here, we have introduced the quantitative criteria. Meanwhile, the concentration of Cl is inversely correlated with Mg. This provides a striking confirmation of our previous studies, as presented in [26,38], where it was shown that the apatite matter in enamel and its variability are strongly stoichiometric. Now, this finding has widened to encompass water and organic matter. Clearly, enamel is the most ordered structure among bioapatites and adheres to strict stoichiometric rules.

All this can be corroborated by the conclusions of contributions by other authors: Pasteris et al. [44], also from the Drouet and Rey group, postulated that the surface layers of apatite crystals are saturated with water; the authors of [45] and the papers by Bertinetti et al. [30], Gordon [46], La Fontaine et al. [47], and DeRocher et al. [48] have shown that tissue cells are surrounded by a layer with enhanced Mg contents. Since the growth in Mg contents is negatively correlated with the presence of Ca (and P), the water and organic matter are in inverted relationship with Ca (Figure 3a,b) and P. One can hypothesize that it is coupled with a delicate change in the character of chemical interactions: the change in Mg^2+^ and OH^−^ ions on Ca^2+^ and Cl^−^ entities forms bonds of clearer ionic character, in contrast to the more covalent primary version. Knowledge on the amount of ions released from apatite-based materials with tunable specific surface area sizes is presented by the authors of [49]; moreover, they assert that the sizes of Nano-HA allow researchers to program hydroxyapatite resorption and create biodegradable bone grafts.

The coupling of organic matter with apatite is more likely to be possible in locations with a more covalent character. Thus, the relationship illustrated in Figure 1a seems to be reasonable. Moreover, strict observation of the dentin–enamel junction (DEJ) [50] allows researchers to estimate that Mg is the first element to arrive in the enamel zone. This suggests that Mg cooperates with amelogenin and enamelin in forming bioapatites. The present study proves that the coupling of Mg with organic matter and water changes occurs in enamel and is quantifiable. To our knowledge, this finding is novel in the literature.

**Figure 7:**
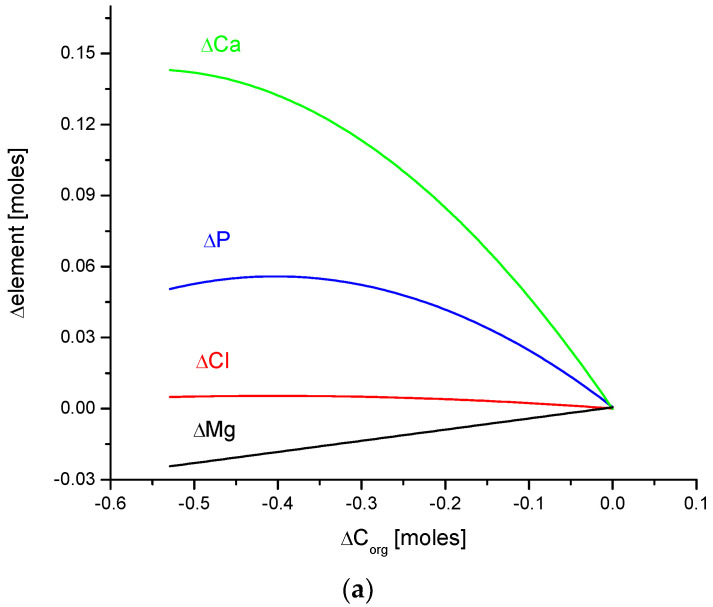

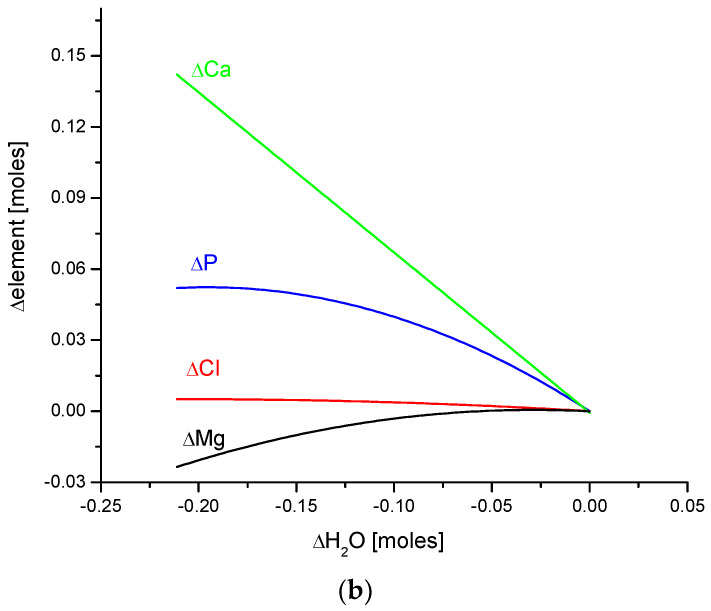
Quantitative coupling of (**a**) organic matter and (**b**) water with the basic elements of enamel bioapatite. The relationships are shown on the molar basis.

In this paper, the clear mutual relationship between Ca, P, Mg, Cl, H_2_O, and organic matter is evident. Figure 7 shows the interrelation of organic matter with ions of the mentioned elements and the same is valid for water levels. All those substances form the main cycle of chemical interactions in enamel bioapatite. These chemical dependencies are related to animal species.

The data obtained in this paper allowed us to widen the illustrative presentation of the variability of elements and basic compounds in the enamel; these are reviewed in the following order: air–enamel surface–dentin–enamel junction (DEJ) on the lingual surface [26]. We assume that the relationship

ΔH_2_O = 18.2ΔMg + 389.4 [ΔMg]^2^ (4)

is universal one and can be applied for the variable Mg contents in the mentioned order.

The data are presented in Table 1. 

If one extracts the approximate stoichiometric coefficients for the described ion exchanges between species from p → o→ b → h sequence (see Figure 1 and Figure 2), then the result is derived as follows:

24ΔCa: 4ΔMg: 1ΔCl

This provides nearly the same result as that for the ion exchange that occurs within human enamel from the dental–enamel junction (DEJ) towards the air–enamel boundary:

25ΔCa: 5ΔMg: 1ΔCl

This is nearly identical to the results for the fragment of apatite, which is sampled during formal transformations; however, the increments of P, CO_3_^2−^, and Na behave in a chaotic manner.

One of the most impressive results is that all kinds of enamels for the four studied organisms lay on one straight line, expressing the relationship between the energy of transformations and the change in the crystallographic parameter, d, or the change in the sinΘ value, treated as an independent variable (Figure 6). This means that the energy of transformations is linearly connected with the changes in universal crystal size in the first case and with momentum transfer in the second case.

## 4. Materials and Methods

### 4.1. Materials

The materials and methods used int his study are supported by our previous research concerning tooth enamel, the literature on the dentin–enamel junction (DEJ) and bones [38,39,51], and by the data available in papers written by the team from the University of Murcia, Spain [40,52]. Importantly, the data were used because they covered several mammal species: bovine, ovine, porcine, and human teeth. The ionic/compound contents (Ca, P, Mg, Na, CO_3_^2−^, H_2_O, organic C) were of interest in our approach and the data were available in the mentioned Spanish publications. It is important to note that these studies analyzed—among others—the water contents of the enamels. Both the applied technique—thermogravimetric analysis with a mass spectrometry detector (TG–MS)—and the method of sample preparation allowed the researchers to detect water, except in samples where there were loosely bonded. Moreover, the crystallographic parameters, a and c, for the hexagonal system, were studied. Wider sets of data [53,54] have been published; however, they are not as detailed as those cited here.

### 4.2. Methods

For numerical calculations, Origin9.1 (OriginLab in Northampton, MA, USA) was applied. We used the set of equations described in [26], which presents the interrelationships between the energy of transformation and the universal crystallographic dimension, d, which are as follows:

ΔE = (6.2/d_1_)(1/sinΘ_1_ − 1/sinΘ_2_) (5a)

ΔE = (6.2/sinΘ_1_)(1/d_2_ − 1/d_1_) (5b)

ΔE = −6.2˟d/(d^2^˟sinΘ) (5c)

ΔE = −6.2˟Δ(sinΘ)/(d˟sin^2^Θ) (5d)

ΔE = −(1/6.2)˟Δd˟E^2^˟sinΘ (5e)

The equations are mutually equivalent. The symbols have the following meanings:

E, ΔE—energy and change in energy;
d, Δd—the dimension from Braggs’ equation and its change, respectively;
Θ—the angle between the impinging X-ray and the crystallographic plane.

All the data concerning the elemental amounts/concentrations were recalculated to moles.

All the results were relative since they were compared to the values determined for humans. An important assumption is that interelement relationships were estimated from a statistical point of view; here, we compared the coefficients of determination, R^2^, and estimated that its greater value implies a truer result. If this coefficient is equal to 1, it is supposedly concluded that the dependence is a functional one. The results were, however, also estimated from a functional point of view, i.e., that the polynomial relationship of the smaller order is, as a rule, more important if it concerns the relations between the elements.

## 5. Conclusions

We have observed the detected values of the a and c crystallographic dimensions for the bioapatites in the enamels of four different species, according to a methodology established by Ortiz-Ruiz et al. [40]. The results of our study on the dependence of the energy of transformation on the universal crystallographic dimension, d, and thus on the growth of the volume of the crystallographic cell and the momentum transfer, indicate some strong trajectories; along these, organisms build their own specific kind of bioapatite cell. These results suggest that there is a possibility of steering transformations of animal bioapatites in a given desired direction; alternatively, there is a possibility of synthesizing apatites within assumed parameters. Moreover, the molar amounts of water and organic matter are strongly connected with the amounts of Ca, P, Mg, and Cl, but are positively correlated only with Mg. To our knowledge, these findings are novel in the literature; future research could hopefully confirm our findings using tissues of animals which are more systematically distant than the species mentioned by us. Please note that the experimental base for our study was extremely simple—it is sufficient to determine the chemical amounts and crystallographic parameters of the given bioapatites. It seems that the Mg molar level is a good measure of organic matter and water contents in enamel apatite. Moreover, the quantities of organic matter and water in enamel are also mutually and strictly dependent. On the one hand, these findings can be used in the development of methods of synthesis of substances which are similar to the original enamel; on the other hand, these findings indicate that the introduction of specific amounts of Mg can determine the wetness of bioapatite.

## Figures and Tables

**Table 1:** The amounts of different elements/compounds in different locations of enamel, related to 10,000 Ca atoms.

Element/ Compound	Surface Location	Location Close to DEJ	Mean Values
Ca	10,000	10,000	10,000
P	6451	6389	6420
Mg	25	165	95
Na	162	453	308
CO_3_^2−^	321	748	535
Cl	179	30	105
F	88	---	7
H_2_O	236	1554	895
C_org_	992	6548	3770

Note: C_org_ is organic carbon.

## Data Availability

Not applicable.
